# Successful Application of Deep Learning to Biosecurity Surveillance: A Systematic Approach Using the Asian House Gecko, *Hemidactylus frenatus* as a Case Study

**DOI:** 10.1002/ece3.73456

**Published:** 2026-04-20

**Authors:** André G. deSouza, Grey T. Coupland, Kok Wai Wong, Paul Doughty, Simon J. McKirdy

**Affiliations:** ^1^ Harry Butler Institute Murdoch University Murdoch WA Australia; ^2^ Collections & Research Western Australian Museum Perth WA Australia

**Keywords:** artificial intelligence, identification accuracy, invasive species, rapid response, taxonomic identification

## Abstract

AI‐based solutions offer the potential for rapid taxonomic identification of species of biosecurity concern, enhanced global accessibility and time‐saving in contrast to traditional taxonomic identification by humans. This study provides a systematic approach to the application of deep learning for biosecurity surveillance, using the Asian House Gecko (AHG), *Hemidactylus frenatus* , Schlegel, 1836, as a case study. An effective triage tool for rapid initial identification of this invasive species was developed using machine learning, achieving high accuracy. This demonstrates the efficacy of deep learning for identifying complex morphological characteristics. The AI model used the AHG's head as a key identifying feature, highlighting the importance of specific morphological features for effective identification of target species. A structured approach for the use of machine learning was developed, which included the collation of source images, cataloguing, tagging, naming and storing images, validating and uploading images, labelling images, creating, training and deploying the model, testing model accuracy and retraining the model. This procedure allows for more rapid application of the methodology in biosecurity surveillance. The structured methodology developed can be applied to similar AI‐based projects. Outcomes of this research have the potential to reduce the time delays associated with taxonomic identification of invasive species, allowing follow‐up action to occur sooner. Reducing time delays is critical to implementing effective biosecurity measures.

## Graphical abstract

Invasive gecko species, such as the Asian House gecko, have the potential to threaten native gecko species for food and habitat, when introduced into new areas. We examine the role of artificial intelligence, in rapid detection of this species, to allow early triage of identification of the Asian House gecko, where biosecurity measures are in place for ecological conservation, to reduce the time to deploying resources for further detections and management of the spread of this species, as well as a guiding methodology that can be employed, in developing projects of this nature, where species identification can be undertaken using machine learning techniques, such as image classification.

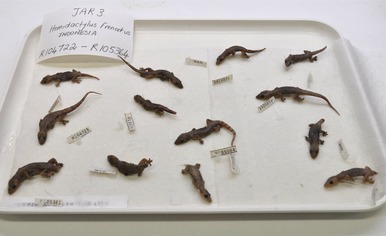

## 1 Introduction

Biosecurity aims to prevent the introduction and spread of disease or invasive plants and animals (Hulme et al. 2023). Early detection of invasive species is critical to controlling and eradicating target organisms, minimising the risk of pests becoming established in new habitats (Hulme 2014). Biosecurity is an important part of government efforts to prevent, respond to and recover from pests and diseases that threaten the economy and environment (Meyerson and Reaser 2003). The substantial increase in global trade and passenger movement has been associated with a rise in incursions and detections of introduced species (Jarrad 2015). These biological invasions can have a devastating impact on the environment and resources, resulting in high remediation costs (Blackburn et al. 2019; Pimentel et al. 2000, 2005). Effective biosecurity surveillance is therefore key to limiting the likelihood of pest species incursions and subsequent establishment (Anderson et al. 2017). Biosecurity surveillance efforts are divided into three broad areas: pre‐border, border and post‐border (Hulme 2014). These efforts are often concentrated at pre‐border and border locations to minimise the potential for an incursion and associated costs (Liu et al. 2024).

We provide a case study in biosecurity management for a potentially invasive gecko species. In this case study, pre‐border and border biosecurity surveillance, along with associated specimen collections, are most commonly conducted manually. Suspect specimens may be collected by biosecurity personnel untrained in morphological taxonomy or members of the general workforce and then submitted for identification by an expert. Untrained workers may err on the side of caution and report common native species, where they lack expertise; this increases the cost of surveillance and can tie up taxonomic specialists with routine identifications. Consequently, the timeframe from surveillance and specimen collection to identification can be both time‐consuming and expensive (Jurdak et al. 2015), especially where specimens are transported to a taxonomist over large distances, as in this case study. However, effective biosecurity management often requires a rapid response once an incursion occurs (Streito et al. 2023), particularly as managing an invasion is often more environmentally and economically costly once a pest/invasive species is established in a new environment (Canyon et al. 2011). Invasive species may resemble native species in basic form and appearance. Due to regulations, taxonomic experts will examine specimens in more detail to discriminate between species, but this often requires laboratory examination to search for subtle diagnostic characters. As such, rapid diagnostic methods can facilitate the early in situ detection of pest species, enabling faster response times to potential incursions (Baxter and Hamilton 2016). This often relies on taxonomic expertise.

Morphological taxonomy is essential to conservation biology (Paquin et al. 2008; Pérez‐del‐Olmo et al. 2011) and biosecurity, as accurate characterisation of species is important to understanding biodiversity and biosecurity efforts, since species may superficially appear similar but exhibit very different behaviours and characteristics from a biosecurity perspective, as noted in this case study. Typical biosecurity regulations also require formal identification of species, with detection of invasive species often triggering biosecurity responses aimed at containing and eradicating the target organism. However, as the availability of expert taxonomists is declining (Hopkins and Freckleton 2002), taxonomic knowledge is in high demand, making it difficult to address identifications promptly. Where taxonomic identification is time‐critical, such as when an invasive species arrives at a border location or port, delays can impede the application of appropriate biosecurity measures, especially when additional time is required to transport the necessary samples for identification (Streito et al. 2023). Rapid and accurate identification of invasive organisms is critical for early detection and management of an incursion (Hulme et al. 2023), and there is now progress towards using automated methods for detecting invasive species to manage these challenges (Leung et al. 2025).

Automated methods for detecting specific species are demonstrably faster and less likely to yield false negatives than manual methods (Hodgson et al. 2018). They are also comparable to human experts (Rogers et al. 2019; Ärje et al. 2020). In some instances, such as detecting medical carcinomas, deep convolutional neural networks (CNNs) achieve performance on par with human experts (Esteva et al. 2017). Progress in machine learning techniques, broadly referred to as artificial intelligence (AI), means automated detection is less resource‐intensive to implement: pre‐existing open‐source algorithms can be retrained to detect specific species or signs of disease with relatively little relevant data (Robin et al. 2024). Machine learning algorithms are being used to classify organisms to a high level of accuracy (Blair et al. 2020, 2022, 2025). There is a strong case for adopting an AI‐based triage tool when expert availability is limited and timely action is required. While AI is not likely to be a suitable solution for every taxonomic project, the benefits of adopting AI‐based triage solutions are likely to speed up decision‐making where an expert is not immediately available.

Deep‐learning image classification tools have the potential to identify complex morphological characteristics that will enhance biosecurity surveillance (Ding and Taylor 2016; Doan 2023; Xia et al. 2018). Deep learning is an AI function that uses artificial neural networks—algorithms that mimic the human brain's data processing and pattern recognition capabilities—for decision‐making. Deep learning is a way of ‘training’ an algorithm to learn how to perform a specific task. Training involves feeding large amounts of data into the algorithm (Taye 2023), which performs a task repeatedly, adjusting each time to improve the outcome. Generally, the greater the amount of data the algorithm receives, the better the algorithm can mimic the way in which humans process information and make decisions (Shorten and Khoshgoftaar 2019).

As the case study discusses, gecko specimens suspected of being non‐indigenous are currently transported long distances for formal identification—a process that can take several days. During this time, a highly mobile species, such as a climbing gecko or frog, could stow away in cargo and travel farther from the original collection site, resulting in increased remediation costs. This research investigates the application of a real‐world deep‐learning biosecurity surveillance system to reduce the time delays associated with traditional taxonomic identification. The case study examines the use of deep learning to identify the invasive gecko species, the Asian House Gecko ( *Hemidactylus frenatus* Schlegel, 1836), which poses a biosecurity threat to a remote island ecosystem. If adopted, an AI‐based solution to identify invasive geckos could facilitate early detection, enabling follow‐up action sooner.

## 2 Materials and Methods

### 2.1 Study Site and Target Organism

**FIGURE 1:**
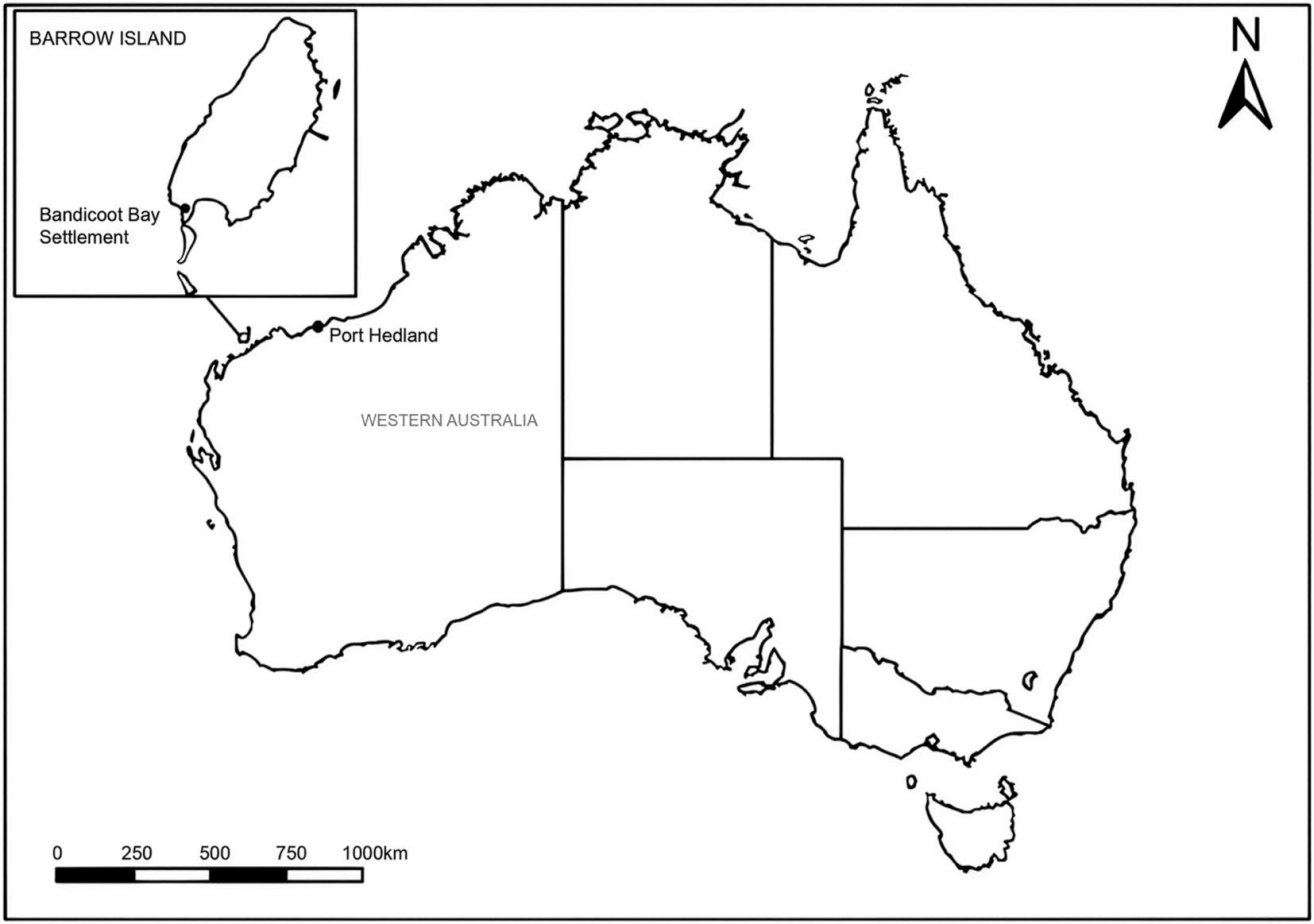
Map showing location of Barrow Island off the north‐west Australian coastline (Image modified from Skippington et al. 2022).

Remote Barrow Island is situated approximately 70 km off the north‐west coast of Australia (Moro and MacAulay 2010) (Figure 1). The island is home to over 24 endemic terrestrial species (Davidovitch et al. 2009), including several gecko species. Its isolation has kept it largely free from unintended introduced animal species. However, the detection of the Asian House Gecko (AHG) on Barrow Island in 2015 (Fitzgerald 2015) highlights the potential of introduced species to impact native gecko populations. On the Australian mainland, the AHG has been linked to the displacement of at least six species of native geckos (Hoskin 2011). The AHG is known to compete with native species for food sources and habitats (Yang et al. 2012) and carry new parasites (Hoskin 2011). Research comparing *Hemidactylus frenatus* with *Hemidactylus garnotii* and *Lepidodactylus lugubris* indicates that the AHG is likely to approach, displace and attack these two native gecko species and is a threat to their hatchlings (Bolger and Case 1992). Consequently, if it were to establish on Barrow Island, the AHG would likely threaten the native species.

Approval for Chevron Australia to build and operate liquefied natural gas (LNG) processing facilities on Barrow Island was subject to stringent plant and animal biosecurity measures (Environmental Protection Authority 2009), including a non‐indigenous species surveillance program (Chevron Australia 2009). If a potential non‐indigenous species, such as the AHG, evades pre‐border biosecurity measures, it is critical that detection and identification measures are in place on Barrow Island to prevent its establishment and spread (Callan et al. 2011). Rapid identification of suspect specimens is key, but the current system requires their transportation to the nearest taxonomist at the Western Australian Museum, a journey of over 1200 km and several days. The delay may allow other undetected individuals on the island to move beyond the initial detection area, requiring a larger search radius for remediation and increased resources.

### 2.2 Identifying Features of Gecko Species and Source Images

In a field situation, the morphological characteristics of the AHG make it difficult for untrained personnel to distinguish it from some native gecko species.

In developing a deep‐learning model using image classification techniques, 49 gecko species were assessed for potential inclusion. Of these, the list was refined to 13 gecko species (including the AHG), some of which share attributes with the AHG (Table 1). On the list were species endemic to Western Australia, particularly the Pilbara region, including Dampier, the closest major port to Barrow Island. In addition, outlier species, morphologically distinct from the AHG, were used to test the capacity of the AI model; these included *Heteronotia binoei* (native to BWI; ground gecko), *Strophurus jeanae* (native to BWI; slender with spindly limbs, no spines on tail) and *Strophurus rankini* (coastal distribution south of BWI; twig mimic but with spines on the tail, similar to AHG) as recommended by the taxonomic expert. The taxonomist relies initially on the spines around the tail of the AHG for identification, and the toepads, which extend further along the length of the gecko's toe, not just the tip, as is typical of *Gehyra*.

**TABLE 1:** List of gecko species photographed to create a deep‐learning model.

Gecko species	Similar to AHG	Distinguishing features
Exotic to Australia:		
*Hemidactylus frenatus*	—	*Spines on tail*
*Hemidactylus garnotii*	Yes	*Flat tail*
*Hemidactylus platyurus*	Yes	*Flat tail*
*Lepidodactylus lugubris*	No	*Cylindrical body*
Endemic to BWI:		
*Strophurus jeanae*	No	Narrow body, spindly limbs
*Heteronotia binoei*	No	Upright posture, low tubercles over body
*Gehyra punctata* ^a^	Yes	Red with spots, no spines on tail, toepads round at tip
*Gehyra variegata*	Yes	No spines on tail, toepads round at tip
Endemic to WA:		
*Christinus marmoratus*	Yes	Thick tail lacking spines
*Gehyra media*	Yes	As for *G. punctata*
*Gehyra polka*	Yes	As for *G. punctata*
*Strophurus rankini*	No	Spines on top of tail only, spindly limbs

^a^ Introduced prior to the year 2000.

The data sources for images used to train the AI model were fragmented. Initial attempts were made to source images from disparate databases by contacting image sources; however, no single source provided more than three to five images of each species. Several of these were of poor quality, of unknown origin, or of uncertain accuracy. Consequently, it was necessary to create an image library of manually captured photographs of specimens from the Western Australian Museum to effectively train the model.

**FIGURE 2:**
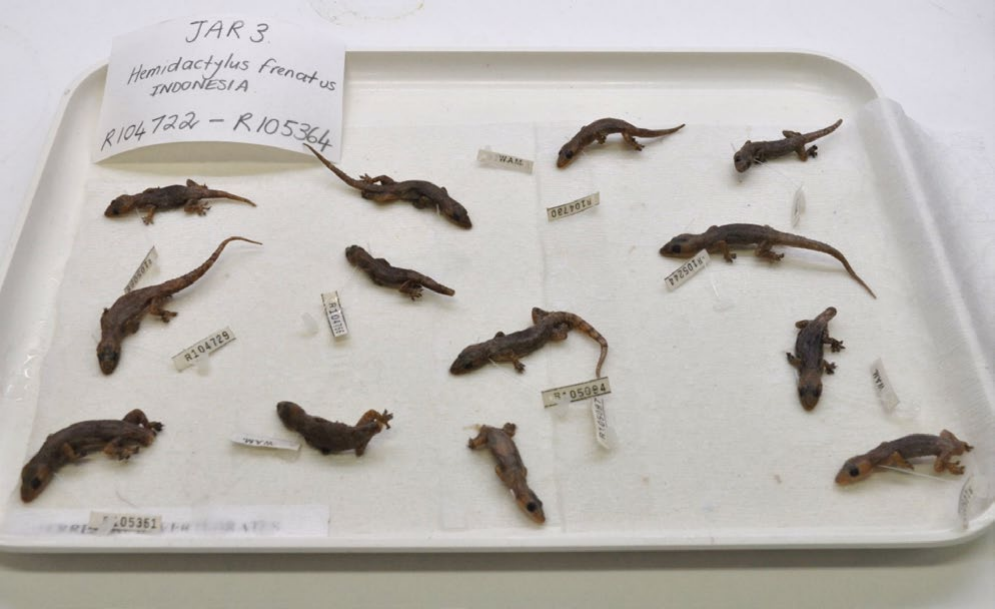
Photograph of 13 Asian House Gecko, *Hemidactylus frenatus* , specimens staged on white museum trays.

To photograph geckos, preserved specimens were removed from ethanol storage jars, staged on white museum trays (Figure 2) and manually photographed using a Nikon DSLR camera, the D5000, set on automatic. White museum trays were selected as a backing to remove unnecessary digital noise and reduce the need for image post‐processing before training the AI model. Staging was done in a manner that replicates the way specimens might be received from the field for identification. Specimens collected on Barrow Island are often stored in ethanol and removed and staged on museum trays for identification.

Several sequential steps were required to train the AI model to detect the target organism, *H. frenatus* :

1. Validate, catalogue and store images:
2. Label and upload images;
3. Create, train and deploy the AI model; and
4. Test model accuracy and retrain the model.

A brief description of each step follows:

### 2.3 Validate, Catalogue and Store Images

At the Western Australian Museum, gecko specimens are identified by an expert herpetologist. This expert (co‐author Paul Doughty) is also the recognised west Australian morphological taxonomist for a range of gecko species endemic to the Pilbara region. Following identification, specimens are stored in a separate jar, each labelled with the species name, with each specimen receiving an archival tag with a unique identification number. The taxonomist uses a combination of published morphology (Uetz et al. 2026), genes (Agarwal et al. 2026), distribution and his own experience. This identification of specimens, and storage in labelled jars, provided the validation of images taken of the specimens, where the photographer staged specimens based on the jar labels. Over 700 photographs taken of these specimens were saved separately to a data drive. They were catalogued into digital folders labelled for each species, based on the jar labels and within each species into used and unused images. This ensured that the images were validated to species level—identified morphologically and/or genetically—so the model training could run on accurately selected images.

### 2.4 Label and Upload Images

Identifying characteristics were determined for the AHG. This included assessing specific morphological traits, including the spur on the AHG tail and the head shape. To train the model, the whole body of the gecko was used rather than individual parts.

For every image, a rectangular bounding box was drawn around the target organism. This bounding box was used by the model to define the target area for inclusion in model analytics and to train the model. Each bounding box was tagged with the species name to identify the geckos. A small subset of images was set aside for model testing. The remaining images were uploaded for model training to the IBM PowerAI Vision platform. IBM provides software titled PowerAI Vision, as a video and image analysis platform built for IBM Power Servers, which uses Graphics Processing Units to optimise performance. PowerAI Vision includes tools and interfaces to label images and videos to train an AI model to classify images or video frames, also known as image classification, or to find objects in images or video frames, which is also known as object detection. In this case study, we explore the use of image classification.

### 2.5 Create, Train and Deploy the AI Model

In this case study, the AI model appears to use the head to identify the AHG. AI models infer the key characteristics from training data that assist in identifying the target. Once trained, the model was deployed for testing. Due to Graphics Processing Unit (GPU) limitations, the PowerAI Vision platform server used was limited to four trained and four deployed models at a time.

### 2.6 Test Model Accuracy and Retrain the Model

The model was tested for accuracy using the subset of images previously validated and reserved for the purpose. Images were selected randomly and not used repeatedly to test the model.

To improve the accuracy of species identification and to increase certainty in the diagnosis, the model was re‐trained following initial testing, using additional labelled images. After six iterations of training the model, the model had sufficient data to provide high confidence in its identification. This occurred until the model correctly identified the 10 images presented for diagnosis with 100% accuracy, and with a 98% mean confidence score for each image—that is, the certainty the model has that its predictions are correct.

## 3 Results

### 3.1 Early‐Stage Model Development and Iterative Process

During the early stages of model development and testing, a low image count of 121 images resulted in poor identification accuracy. This is likely because the number of images did not provide sufficient variability for the highly refined diagnostic features required for morphological identification. The generated heatmaps, showing areas that the AI model was using for identification, were also inconsistent. This informed the methodology, indicating the need for more images.

With a higher image count, accuracy improved. Ten images were used to test the model, with model accuracy at 100%, thus indicating that it could correctly identify the AHG. Table 2 has the results for the 10 images, showing the high ‘accuracy’ or confidence that the model has in its own diagnosis. Table 3 shows the number of data points (photographed specimens) of each species needed to achieve this accuracy.

**TABLE 2:** Declared model accuracy for each Asian House Gecko test image (*n* = 10).

Image 1	99%	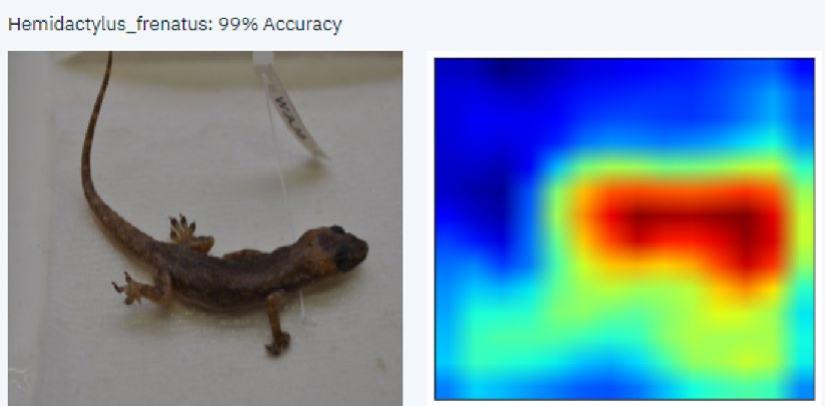
Image 2	99%	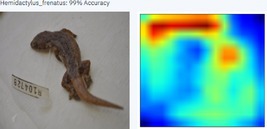
Image 3	99%	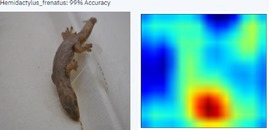
Image 4	100%	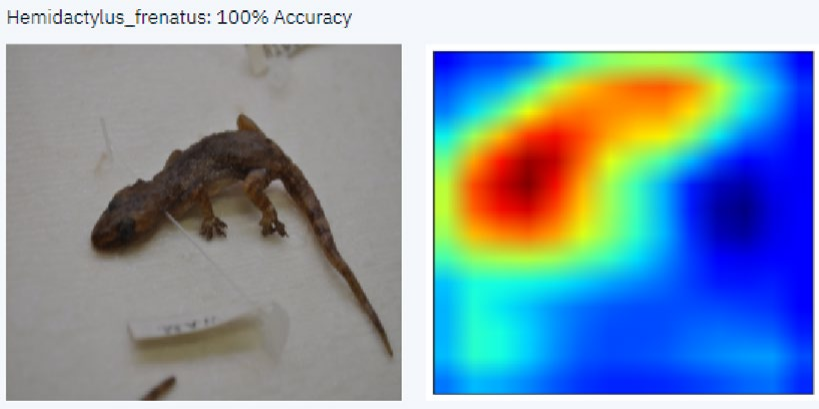
Image 5	99%	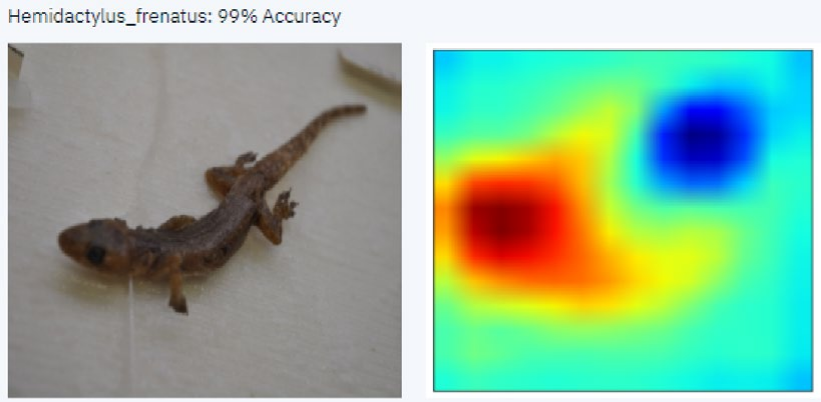
Image 6	92%	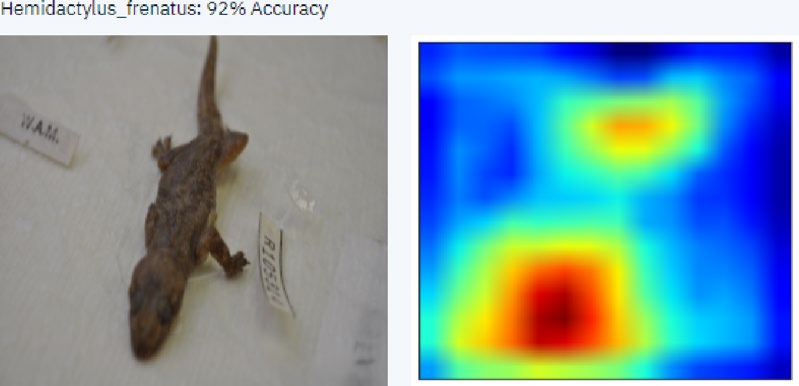
Image 7	99%	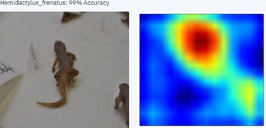
Image 8	91%	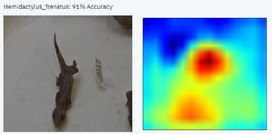
Image 9	98%	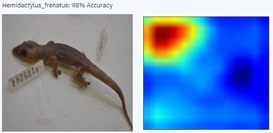
Image 10	100%	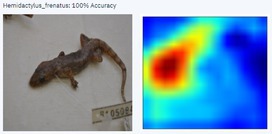
Mean	98%	

**TABLE 3:** Number of data points (all specimens) required to train a model to reach 100% accuracy from 10 tested images.

Gecko species	Number of labelled data points (specimens)
*Hemidactylus frenatus*	368
*Gehyra pilbara*	155
*Gehyra punctata*	172
*Gehyra variegata*	78
*Heteronotia binoei*	282
*Gehyra media*	9
*Gehyra polka*	20
*Hemidactylus garnotii*	58
*Hemidactylus platyurus*	48
*Christinus marmoratus*	174
*Strophurus rankini*	95
*Lepidodactylus lugubris*	29
*Strophurus jeanae*	101

Table 2 includes screenshots of the results of each of the 10 images presented to the AI model for identification. In this instance, the screenshots show the species name the model has identified, the model accuracy (confidence in its diagnosis), a thumbnail of the original image presented for diagnosis and a heatmap side by side showing the parts of the image that the model is using for diagnosis. Red colouration identifies the parts that are highly relevant for diagnosis; the parts strongly being used for diagnosis taper off to orange and yellow, and blue indicates the parts of the image that are not valuable for identification.

### 3.2 Identifying Characteristics of Gecko Species

Thirteen gecko species morphologically similar to the AHG were used to train the AI model. From these species, the AI used several morphological features to distinguish the various species. In 8 out of 10 instances, as shown in Table 2, the AI used the head region to identify the gecko species. This might highlight new morphological characteristics not previously considered for the identification of this species or may be unknowingly used by human taxonomists.

## 4 Discussion

This study developed a triage tool for rapid initial identification of invasive species using machine learning with a high degree of accuracy (100%). The results suggest that, where existing expertise is limited or unavailable, this methodology provides practitioners with a detailed process for developing an AI‐based solution with immediate applications in biosecurity surveillance. AI‐based solutions offer novelty, global access and are potentially time‐saving when compared to human taxonomic identification. Additionally, taxonomists trained in morphological identification spend years developing their skills, at considerable cost. Taxonomists are typically subject matter experts. With the assistance of trained taxonomists, in contrast, AI models could be trained to identify a wide range of organisms in a far shorter amount of time. In this study, the AI model appeared to use the head for identifying the AHG, perhaps highlighting that this is also an important, yet unacknowledged, morphological feature.

**FIGURE 3:**
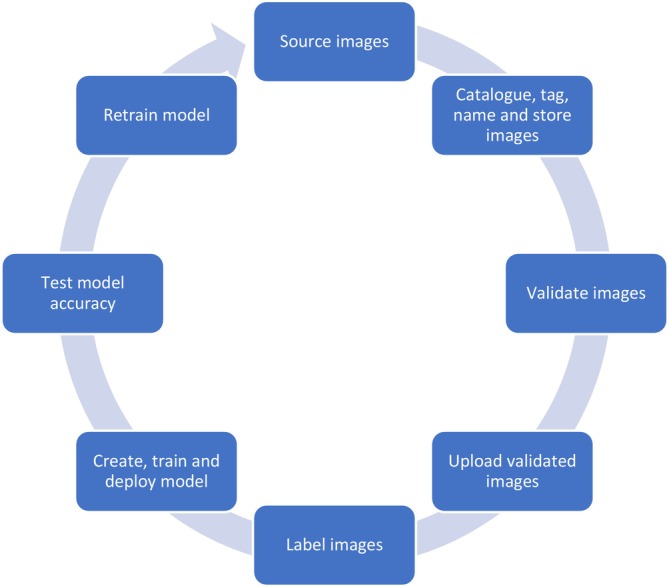
Systematic procedure to create an effective model for biosecurity surveillance purposes using deep learning.

Developing an AI model to successfully classify target organisms using image recognition requires a sequence of specific steps to ensure model accuracy. In the case of the AHG, the model was developed following a structured methodology (Figure 3). Adopting this approach is likely to benefit other practitioners in developing similar AI‐based projects.

## 5 Systematic Procedure to Create an Effective Model for Biosecurity Surveillance Purposes Using Deep Learning

### 5.1 STEP 1: Importance of Source Images

Understanding the types of images needed is important for model development. Firstly, the images must provide sufficient detail to accurately identify the target species and its morphological characteristics. Secondly, they need to provide reliable reference material to accurately identify and eliminate species commonly mistaken for the target species.

In deep‐learning contexts, some organisms may be difficult to identify (Berg and Forsyth 2006). This is a consequence of many AI models being poorly equipped to handle the large morphological variations across organisms, including differences in appearance and aspect (Berg and Forsyth 2006). Organisms that lack distinctive skin or hair texture patterns can be particularly difficult to distinguish for human taxonomists as well (Zeppelzauer 2013). Consequently, where there is suitable variation within the species, a selection of images that represents a broad cross‐section of the target is required to build an accurate model. For example, the image library should include individuals with different colour morphs, morphological variations in body structure (including longer‐than‐typical legs or broader head widths), and images that capture all life stages of the target, if appropriate. In this study, gecko images included individuals missing their tails, as certain gecko species can ‘drop’ their tails to distract predators when threatened (Nagumantri et al. 2021).

Sourcing images for this study began by listing the targeted species for biosecurity surveillance, along with similar species that may be found in the target and surrounding areas. In addition, a range of outlier specimen images was also acquired to provide the AI model with sufficient context. Providing context to an image classification AI model may ensure the model is able to establish relationships between different gecko species (Wang and Zhu 2023). AI models benefit from context to reduce hallucinations and false positives (Galleguillos and Belongie 2010). In addition, if specific identifying characteristics are used to aid identification, such as spines on arachnid legs (Framenau et al. 2022), images should have sufficient detail to reflect these characteristics. A subset of images may include only these characterising features. This could be used by labelling those specific sections of the image to highlight these characteristics during model training.

When collecting images, it is important to note that having multiple images of the same individual with minor posture changes is unlikely to be of significant value for training the AI model. AI models that have been trained with limited or mainly homogenous datasets can be prone to performing well on training data but fail to generalise to new data (Kavzoglu 2009). Variety is key; however, typically, when images are received from a source, a large number may be of the same specimen. If, in a group of 50 images, the actual number of individuals is only 10, the result is limited new information for the AI. Depending on the targeted species in biosecurity surveillance, image availability can be challenging, as there may be only a small number of images per species.

The AI model should be provided with context images likely to be used in the final application. If the AI is required to characterise species based on images taken using a microscope, as opposed to a DSLR camera, then the AI model needs training in microscope images to improve the chances for success. Similarly, if the characterisation is based on species preserved in ethanol, such as in a museum collection, then the AI model requires specific recognition training as ethanol changes the visual characteristics of specimens. In some instances, data may be received pre‐labelled by subject matter experts. To ensure that the model receives training in accurate data, it is also necessary to include human categorisation of images.

When photographing specimens, several points should be kept in mind. For those stored in ethanol, note that there may be colour loss. Specimens may also dry out, making the finer characteristics more difficult to identify, especially in a photograph. If specimens are from a museum collection, they may also have parts removed, incisions that expose internal organs, or labels or tags affixed.

Setting up specimens for photography during the study was a careful process. Specimens were adequately separated in multiple stages, with no overlap, for easier labelling. Specimen labels were excluded where possible. Some AI interfaces may only use rectangular labelling, rather than free‐form shape labelling. Attempts should be made to vary the background to include colour and texture where appropriate. However, in this case study, the use of the white museum trays was repetitive as this followed the regular process adopted during morphological identification. Furthermore, the study avoided macro photography, as it led to some body parts being blurred.

### 5.2 Step 2: Catalogue, Tag, Name and Store Images

When dealing with large volumes of data, errors are always a possibility. Problems may occur with the incorrect categorising and labelling of images in the AI. As such, it is imperative to understand the importance of using image cataloguing and database software rather than setting up a series of separate folders. For instance, Adobe Lightroom provides a systematic approach to cataloguing images. Also, having a logging system for images uploaded to the AI platform prevents duplicates, thereby reducing potential waste or bias. For example, if images are received from a range of sources with a variety of file names, such as P150, IMG00562, keeping these original file names intact prevents duplicates from being stored.

Maintaining a log of images uploaded to the AI requires ordering the original image names. If they are not sequential or do not follow a fixed nomenclature, it would then be necessary to ‘rename’ the files. ‘Renaming’ the file is achieved by adding a series of tags, including a new name tag (following a fixed, sequential nomenclature), a tag for the name of the image source, location tags (where available) a tag to indicate whether the species in the image has been correctly identified (validated), and the name of the person providing this validation for record purposes. This tagging is best achieved using an image database and cataloguing software.

### 5.3 Step 3: Validate Images

The images uploaded must have been correctly identified or validated by a trained taxonomist to ensure the AI receives high‐quality and accurate information. Inaccurate image labelling may lead to incorrect results (Nigam et al. 2020). The accuracy of the eventual AI knowledge depends on the information used to build the knowledge base. Image validation enables AI to be trained to a high level of accuracy—algorithms need to be able to disregard certain images.

The accuracy level should align with the model's purpose. It is recommended that AI models be used only as part of decision‐making processes and not as a critical tool. In some instances, a low percentage of accuracy may be acceptable, such as when estimating animal counts. Other instances may require a higher level of accuracy, as more resources—for instance, additional surveillance—may be deployed based on an AI‐based diagnosis.

### 5.4 Step 4: Upload Validated Images

Before uploading images, set aside a small subset for model testing. Some AI platforms may offer a bulk upload tool.

### 5.5 Step 5: Label Images

Labelling or tagging images is the provision of ‘training’ on elements of the image that are important to machine learning model development. When labelling images to train an AI model, consideration should be given to identifying characteristics. Whole organisms may be required for species recognition in biosecurity surveillance. In other instances, certain body parts may reveal vital clues about the specific species, along with close‐ups of key identifying characteristics such as tails, foot pads, or antennae. Images may be labelled to identify the species itself—for example, *Hemidactylus frenatus* , *Gehyra punctata* , *Hemidactylus garnotii* , etc.—or using specific body part references. In a place like Australia, where gecko taxonomy is an active area (e.g., Doughty et al. 2018; Kealley et al. 2018), recording individual specimen numbers is also advised, as taxonomic name changes can be tracked accurately.

### 5.6 Step 6: Create, Train and Deploy Model

Once there are enough images, models can be trained. To supplement images, data can also be augmented using features to sharpen, blur, rotate and skew where available. Initially, this case study used data augmentation to increase the number of data points; however, additional data collection meant this was unnecessary.

It is important to consider the model's accessibility, such as via a web interface. One option is to deploy the model on a chip for real‐time use. Model deployment will depend on how the model is intended to be used; for example, via a smartphone, smart glasses, or other technologies.

Once trained, models are deployed for testing; however, there are limiting factors. For this study, only four trained and four deployed models could be used at a time due to GPU (Graphics Processing Unit) limitations. Time is often a restriction when training AI. Rapid model training requires considerably more computational power than slower training.

### 5.7 Step 7: Test Model Accuracy

Testing model accuracy can be achieved using the subset of images set aside for this purpose. It is important to have validated images for model testing so that the species is known and verified. A traditional taxonomist had verified the images used in this study.

### 5.8 Step 8: Retrain the Model

Once the model has been tested and shows low accuracy, more images can be added to improve identification. The process can repeat, leading to the model being retrained. Retraining continues to improve identification accuracy and increases diagnostic certainty.

## 6 Conclusion

Artificial Intelligence (AI) tools provide contemporary society with ways to expedite repetitive or mundane tasks. The study offers insight into using image classification models to detect an invasive animal pest species—the Asian House Gecko, *Hemidactylus frenatus* —where rapid responses are important for managing its spread. The methodology can be adapted to similar work across other species. Future work could include deploying the model to wearables or devices, such as phones, to assist with in‐field capture and immediate triage of this pest species. The study recommends that AI‐based image classification tools not be used for ultimate decision‐making. Instead, they are better as a triage tool or to eliminate those images that are clearly not of the target specimen.

In this case study, as we have observed from the screenshots in Table 2, the AI model appears to be mainly using the head of the AHG to make its identification, whereas the expert taxonomist uses the spines on its tail and other morphological features, such as its toepads, as the first characters to consult for unambiguous identification. This could signal additional morphological features and indeed a potential new methodology for morphological taxonomy for a separate study, where trained AI models and trained taxonomists concomitantly determine the morphological features for species identification.

The development of this model occurred on a server as a paid service. Where costs are a factor, open source and limited free AI servers offer tools for the community and citizen science to become involved in biosecurity management and surveillance, such as in the case of regional biosecurity groups.

## Author Contributions

**André G. deSouza:** conceptualization (lead), data curation (lead), formal analysis (lead), funding acquisition (supporting), investigation (lead), methodology (lead), project administration (lead), resources (equal), software (equal), supervision (supporting), validation (lead), visualization (lead), writing – original draft (lead), writing – review and editing (equal). **Grey T. Coupland:** conceptualization (supporting), supervision (equal), writing – review and editing (supporting). **Kok Wai Wong:** conceptualization (supporting), methodology (supporting), supervision (equal), writing – review and editing (supporting). **Paul Doughty:** conceptualization (supporting), investigation (supporting), resources (equal), writing – review and editing (supporting). **Simon J. McKirdy:** conceptualization (supporting), funding acquisition (lead), resources (lead), software (lead), supervision (lead), writing – review and editing (supporting).

## Funding

This research was supported by an Australian Government Research Training Program (RTP) Scholarship doi.org/10.82133/C42FK220.

## Conflicts of Interest

The authors declare no conflicts of interest.

## Data Availability

Data is available at the following DOI: https://doi.org/10.60867/00000035.

## References

[ece373456-bib-0001] Agarwal, A. , M. Thomas , Y. Hitchen , et al. 2026. “A New Molecular Tool for Detection of the Highly Invasive Gecko, *Hemidactylus frenatus* .” PLoS One 21, no. 2: 18. 10.1371/journal.pone.0338377. PMC12871987 41637447

[ece373456-bib-0002] Anderson, C. , S. Low‐Choy , P. Whittle , et al. 2017. “Australian Plant Biosecurity Surveillance Systems.” Crop Protection 100: 8–20. 10.1016/j.cropro.2017.05.023.

[ece373456-bib-0003] Ärje, J. , J. Raitoharju , A. Losifidis , et al. 2020. “Human Experts vs. Machines in Taxa Recognition.” Signal Processing: Image Communication 87: 115917. 10.1016/j.image.2020.115917.

[ece373456-bib-0004] Baxter, P. W. J. , and G. Hamilton . 2016. “An Analysis of Future Spatiotemporal Surveillance for Biosecurity. Plant Biosecurity CRC, Brisbane.”

[ece373456-bib-0005] Berg, T. L. , and D. A. Forsyth . 2006. “Animals on the Web. 2006 IEEE Computer Society Conference on Computer Vision and Pattern Recognition (CVPR’06). 2, 1463–1470.” 10.1109/CVPR.2006.57.

[ece373456-bib-0006] Blackburn, T. M. , C. Bellard , and A. Ricciardi . 2019. “Alien Versus Native Species as Drivers of Recent Extinctions.” Frontiers in Ecology and the Environment 17, no. 4: 203–207. 10.1002/fee.2020.

[ece373456-bib-0053] Blair, J. D. , K. Khidas , and K. E. Marshall . 2025. “Leveraging Synthetic Data Produced From Museum Specimens to Train Adaptable Species Classification Models.” PLoS One 20, no. 9: e0329482. 10.1371/journal.pone.0329482. 40901908 PMC12407421

[ece373456-bib-0007] Blair, J. , M. D. Weiser , K. de Beurs , M. Kaspari , C. Siler , and K. E. Marshall . 2022. “Embracing Imperfection: Machine‐Assisted Invertebrate Classification in Real‐World Datasets.” Ecological Informatics 72: 101896. 10.1016/j.ecoinf.2022.101896.

[ece373456-bib-0052] Blair, J. , M. D. Weiser , M. Kaspari , M. Miller , C. Siler , and K. E. Marshall . 2020. “Robust and Simplified Machine Learning Identification of Pitfall Trap‐Collected Ground Beetles at the Continental Scale.” Ecology and Evolution 10: 13143–13153. 10.1002/ece3.6905. 33304524 PMC7713910

[ece373456-bib-0008] Bolger, D. T. , and T. J. Case . 1992. “Intra‐ and Interspecific Interference Behaviour Among Sexual and Asexual Geckos.” Animal Behaviour 44, no. 1: 21–30. 10.1016/S0003-3472(05)80750-X.

[ece373456-bib-0009] Callan, S. K. , J. D. Majer , K. Edwards , and D. Moro . 2011. “Documenting the Terrestrial Invertebrate Fauna of Barrow Island, Western Australia.” Australian Journal of Entomology 50, no. 4: 323–343. 10.1111/j.1440-6055.2011.00818.x.

[ece373456-bib-0010] Canyon, D. , I. Naumann , R. Speare , and K. Winkel . 2011. “Environmental and Economic Costs of Invertebrate Invasions in Australia.” In Biological Invasions: Economic and Environmental Costs of Alien Plant, Animal, and Microbe Species, edited by D. Pimentel , 2nd ed. CRC Press. 10.1201/b10938.

[ece373456-bib-0011] Chevron Australia . 2009. “Gorgon Gas Development and Jansz Feed Gas Pipeline; Terrestrial and Marine Quarantine Management System.” https://australia.chevron.com/‐/media/australia/our‐businesses/documents/terrestrial‐and‐marine‐quarantine‐management‐system.pdf.

[ece373456-bib-0012] Davidovitch, L. , R. Stoklosa , J. Majer , et al. 2009. “Info‐Gap Theory and Robust Design of Surveillance for Invasive Species: The Case Study of Barrow Island.” Journal of Environmental Management 90, no. 8: 2785–2793. 10.1016/j.jenvman.2009.03.011. 19386410

[ece373456-bib-0013] Ding, W. , and G. Taylor . 2016. “Automatic Moth Detection From Trap Images for Pest Management.” Computers and Electronics in Agriculture 123: 17–28. 10.1016/j.compag.2016.02.003.

[ece373456-bib-0014] Doan, T. N. 2023. “Large‐Scale Insect Pest Image Classification.” Journal of Advances in Information Technology 14, no. 2: 328–341. 10.12720/jait.14.2.328-341.

[ece373456-bib-0015] Doughty, P. , M. Pepper , A. M. Bauer , and J. S. Keogh . 2018. “Spots Before the Eyes: Revision of the *Gehyra punctata* Species Complex From Western Australia.” Records of the Western Australian Museum 33: 1–50. 10.18195/issn.0312-3162.33(1).2018.001-050.

[ece373456-bib-0016] Environmental Protection Authority, Government of Western Australia . 2009. “Statement that a Proposal may be Implemented‐ Gorgon gas Development Revised and Expanded Proposal: Barrow Island Nature Reserve. Ministerial Statement No. 800. 10 August 2009. Perth, Western Australia.” https://www.epa.wa.gov.au/sites/default/files/Ministerial_Statement/00800.pdf.

[ece373456-bib-0017] Esteva, A. , B. Kuprel , R. A. Novoa , et al. 2017. “Dermatologist‐Level Classification of Skin Cancer With Deep Neural Networks.” Nature 542: 115–118. 10.1038/nature21056. 28117445 PMC8382232

[ece373456-bib-0018] Fitzgerald, B. 2015. “Invasive species Asian house gecko discovered on Barrow Island, posing “significant risk” to the Class A Nature Reserve Australian Broadcasting Corporation.” https://www.abc.net.au/news/rural/2015‐09‐03/asian‐house‐gecko‐found‐on‐barrow‐island/6744724.

[ece373456-bib-0019] Framenau, V. W. , P. de S. Castanheira , and C. J. Vink . 2022. “Taxonomy and Systematics of the New Australo‐Pacific Orb‐Weaving Spider Genus *Socca* (Araneae: Araneidae).” New Zealand Journal of Zoology 49, no. 4: 263–334. 10.1080/03014223.2021.2014899.

[ece373456-bib-0020] Galleguillos, C. , and S. Belongie . 2010. “Context Based Object Categorization: A Critical Survey.” Computer Vision and Image Understanding 114, no. 6: 712–722. 10.1016/j.cviu.2010.02.004.

[ece373456-bib-0021] Hodgson, J. C. , R. Mott , S. M. Baylis , et al. 2018. “Drones Count Wildlife More Accurately and Precisely Than Humans.” Methods in Ecology and Evolution 9, no. 5: 1160–1167. 10.1111/2041-210X.12974.

[ece373456-bib-0022] Hopkins, G. W. , and R. P. Freckleton . 2002. “Declines in the Numbers of Amateur and Professional Taxonomists: Implications for Conservation.” Animal Conservation 5, no. 3: 245–249. 10.1017/S1367943002002299.

[ece373456-bib-0023] Hoskin, C. J. 2011. “The Invasion and Potential Impact of the Asian House Gecko ( *Hemidactylus frenatus* ) in Australia.” Austral Ecology 36, no. 3: 240–251. 10.1111/j.1442-9993.2010.02143.x.

[ece373456-bib-0024] Hulme, P. E. 2014. “An Introduction to Plant Biosecurity: Past, Present and Future.” In The Handbook of Plant Biosecurity: Principles and Practices for the Identification, Containment and Control of Organisms That Threaten Agriculture and the Environment Globally, edited by G. Gordh and S. McKirdy , 1–25. Springer. 10.1007/978-94-007-7365-3_1.

[ece373456-bib-0025] Hulme, P. E. , J. R. Beggs , R. N. Binny , et al. 2023. “Emerging Advances in Biosecurity to Underpin Human, Animal, Plant, and Ecosystem Health.” iScience 26, no. 9: 107462. 10.1016/j.isci.2023.107462. 37636074 PMC10450416

[ece373456-bib-0026] Jarrad, F. 2015. “Introduction to Biosecurity Surveillance: Quantitative Approaches.” In Biosecurity Surveillance: Quantitative Approaches, edited by F. Jarrad , S. Low‐Choy , and K. Mengersen , 1–6. CAB International.

[ece373456-bib-0027] Jurdak, R. , A. Elfes , B. Kusy , et al. 2015. “Autonomous Surveillance for Biosecurity.” Trends in Biotechnology 33, no. 4: 201–207. 10.1016/j.tibtech.2015.01.003. 25744760

[ece373456-bib-0028] Kavzoglu, T. 2009. “Increasing the Accuracy of Neural Network Classification Using Refined Training Data.” Environmental Modelling & Software 24, no. 7: 850–858. 10.1016/j.envsoft.2008.11.012.

[ece373456-bib-0029] Kealley, L. , P. Doughty , P. Pepper , J. S. Keogh , M. Hillyer , and J. Huey . 2018. “Conspicuously Concealed: Revision of the Arid Clade of the *Gehyra variegata* (Gekkonidae) Species Group in Western Australia Using an Integrative Molecular and Morphological Approach, With the Description of Five Cryptic Species.” PeerJ 6: e5334. 10.7717/peerj.5334. 30038877 PMC6054870

[ece373456-bib-0030] Leung, F. K. W. , L. Schwarzkopf , and S. Allen‐Ankins . 2025. “Advancing Invasive Species Monitoring: A Free Tool for Detecting Invasive Cane Toads Using Continental‐Scale Data.” Ecological Informatics 89: 103172. 10.1016/j.ecoinf.2025.103172.

[ece373456-bib-0031] Liu, Y. , P. Wang , G. T. Coupland , M. L. Thomas , D. Zheng , and S. McKirdy . 2024. “Cost‐Effective Portfolio Allocation Across Quarantine, Surveillance and Eradication Using Info‐Gap Theory.” Journal of Applied Ecology 61, no. 10: 2538–2548. 10.1111/1365-2664.14762.

[ece373456-bib-0032] Meyerson, L. A. , and J. K. Reaser . 2003. “Bioinvasions, Bioterrorism, and Biosecurity.” Frontiers in Ecology and the Environment 1, no. 6: 307–314. 10.2307/3868091.

[ece373456-bib-0033] Moro, D. , and I. MacAulay . 2010. A Guide to the Reptiles and Amphibians of Barrow Island. Chevron Australia Pty Ltd.

[ece373456-bib-0034] Nagumantri, S. P. , S. Banu , and M. M. Idris . 2021. “Transcriptomic and Proteomic Analysis of *Hemidactylus frenatus* During Initial Stages of Tail Regeneration.” Scientific Reports 11: 3675. 10.1038/s41598-021-83283-0. 33574494 PMC7878758

[ece373456-bib-0035] Nigam, N. , T. Dutta , and H. P. Gupta . 2020. “Impact of Noisy Labels in Learning Techniques: A Survey.” In Advances in Data and Information Sciences. Lecture Notes in Networks and Systems, edited by M. Kolhe , S. Tiwari , M. Trivedi , and K. Mishra , vol. 94. Springer. 10.1007/978-981-15-0694-9_38.

[ece373456-bib-0036] Paquin, P. , N. Dupérré , J. C. Cokendolpher , K. White , and M. Hedin . 2008. “The Fundamental Importance of Taxonomy in Conservation Biology: The Case of the Eyeless *Cicurina bandida* (Araneae: Dictynidae) of Central Texas, Including New Synonyms and the Description of the Male of the Species.” Invertebrate Systematics 22, no. 2: 139–149. 10.1071/IS07044.

[ece373456-bib-0037] Pérez‐del‐Olmo, A. , S. Morand , J. A. Raga , and A. Kostadinova . 2011. “Abundance–Variance and Abundance–Occupancy Relationships in a Marine Host–Parasite System: The Importance of Taxonomy and Ecology of Transmission.” International Journal for Parasitology 41, no. 13: 1361–1370. 10.1016/j.ijpara.2011.09.003. 22051400

[ece373456-bib-0038] Pimentel, D. , P. Hepperly , J. Hanson , D. Douds , and R. Seidel . 2005. “Environmental, Energetic, and Economic Comparisons of Organic and Conventional Farming Systems.” Bioscience 55, no. 7: 573–582. 10.1641/0006-3568(2005)055[0573:EEAECO]2.0.CO;2.

[ece373456-bib-0039] Pimentel, D. , L. Lach , R. Zuniga , and D. Morrison . 2000. “Environmental and Economic Costs of Nonindigenous Species in the United States.” Bioscience 50, no. 1: 53–65. 10.1043/0006-3568(2000)050(0053:EAECON)2.3.CO;2.

[ece373456-bib-0040] Robin, R. , R. Gualtieri , G. La Scala , and K. P. Barbe . 2024. “Artificial Intelligence in the Diagnosis and Management of Appendicitis in Pediatric Departments: A Systematic Review.” PubMed 34, no. 5: 385–391. 10.1055/a-2257-5122. 38290564

[ece373456-bib-0041] Rogers, T. W. , N. Jaccard , F. Carbonaro , et al. 2019. “Evaluation of an AI System for the Automated Detection of Glaucoma From Stereoscopic Optic Disc Photographs: The European Optic Disc Assessment Study.” Eye 33: 1791–1797. 10.1038/s41433-019-0510-3. 31267086 PMC7002599

[ece373456-bib-0042] Shorten, C. , and T. M. Khoshgoftaar . 2019. “A Survey on Image Data Augmentation for Deep Learning.” Journal of Big Data 6: 60. 10.1186/s40537-019-0197-0. PMC8287113 34306963

[ece373456-bib-0050] Skippington, J. , T. Manne , A. Paterson , et al. 2022. “Macropod Bone Apatite Isotopic Analysis as Evidence for Recent Environmental Change at Bandicoot Bay Pearling Camp, Barrow Island, Australia.” International Journal of Historical Archaeology 26: 1054–1071. 10.1007/s10761-021-00640-5.

[ece373456-bib-0043] Streito, J. , E. Mendes , E. Sanquer , et al. 2023. “Incursion Preparedness, Citizen Science and Early Detection of Invasive Insects: The Case of *Aleurocanthus spiniferus* (Hemiptera, Aleyrodidae) in France.” Insects 14, no. 12: 916. 10.3390/insects14120916. 38132590 PMC10744011

[ece373456-bib-0044] Taye, M. M. 2023. “Understanding of Machine Learning With Deep Learning: Architectures, Workflow, Applications and Future Directions.” Computers 12, no. 5: 91. 10.3390/computers12050091.

[ece373456-bib-0045] Uetz, P. , J. Hallermann , and J. Hosek . 2026. “Hemidactylus frenatus DUMÉRIL & BIBRON, 1836. from The Reptile Database.” https://reptile‐database.reptarium.cz/.

[ece373456-bib-0046] Wang, X. , and Z. Zhu . 2023. “Context Understanding in Computer Vision: A Survey.” Computer Vision and Image Understanding 229: 103646. 10.1016/j.cviu.2023.103646.

[ece373456-bib-0047] Xia, D. , P. Chen , B. Wang , J. Zhang , and C. Xie . 2018. “Insect Detection and Classification Based on an Improved Convolutional Neural Network.” Sensors 18, no. 12: 4169. 10.3390/s18124169. 30486481 PMC6308804

[ece373456-bib-0048] Yang, D. , E. González‐Bernal , M. Greenlees , and R. Shine . 2012. “Interactions Between Native and Invasive Gecko Lizards in Tropical Australia.” Austral Ecology 37, no. 5: 592–599. 10.1111/j.1442-9993.2011.02319.x.

[ece373456-bib-0049] Zeppelzauer, M. 2013. “Automated Detection of Elephants in Wildlife Video.” EURASIP Journal on Image and Video Processing 2013: 46. 10.1186/1687-5281-2013-46. 25902006 PMC4398987

